# Molecular Cloning and Functional Analysis of CTL14 and PPO4: Their Roles in Development, Reproduction, and Antiviral Defense in *Hyphantria cunea*

**DOI:** 10.3390/insects17090885

**Published:** 2026-08-24

**Authors:** Arina Nur Faidah, Tianxing Lu, Qinghong Wang, Lili Sun, Chuanwang Cao

**Affiliations:** Key Laboratory of Sustainable Forest Ecosystem Management-Ministry of Education, Northeast Forestry University, Harbin 150040, China; arina.nurfaidah@yahoo.com (A.N.F.); 15547110324@163.com (T.L.); 13519709029@163.com (Q.W.)

**Keywords:** *Hyphantria cunea*, RNAi, nucleopolyhedrovirus, C-type lectin, prophenoloxidase

## Abstract

The insect innate immune system serves as the primary line of defense against pathogens, regulated by pattern recognition receptors (PRRs) such as C-type lectins (CTLs) and effector systems like prophenoloxidase (PPO). This study aims to provide molecular and functional characterization of the *CTL14* and *PPO4* genes from *Hyphantria cunea*. Cloning results revealed that *CTL14* encodes 378 amino acids, whereas *PPO4* encodes 681 amino acids. Expression analysis indicated that *CTL14* is highly expressed during the fourth instar and within midgut tissues, while *PPO4* expression is predominant during the first instar and in the epidermis. Functional assays demonstrated that silencing both genes significantly increased larval mortality following infection with the NPV virus using RNA interference (RNAi), with mortality rates reaching 75–100% by the tenth day. Furthermore, knockdown of these genes resulted in noticeable changes in larval physiology, manifested by altered feeding behavior and significantly reduced body weight gain. Conversely, the injection of CTL14 protein resulted in a marked increase in larval body mass accumulation compared to controls. These findings underscore the crucial roles of *CTL14* and *PPO4* as they are involved in both the antiviral defense system and the physiological development of *H. cunea*, thereby identifying them as potential novel targets for biological pest control strategies.

## Simple Summary

The fall webworm (*Hyphantria cunea*) is a destructive invasive pest that causes serious damage to forest and agricultural plants. Understanding its immune system is important for developing effective and environmentally friendly control strategies. In this study, we investigated the roles of two immune-related genes, *CTL14* and *PPO4*, in the larval development, antiviral defense, and reproduction of *H. cunea*. Silencing these genes via RNA interference significantly reduced larval survival, growth, and resistance to nucleopolyhedrovirus infection. Moreover, gene silencing led to severe reductions in adult fecundity and egg hatchability, especially after *CTL14* suppression. Conversely, injection of purified immune proteins improved larval fitness and growth efficiency. These findings demonstrate that *CTL14* and *PPO4* are essential for both immunity and development in *H. cunea* and highlight their potential as molecular targets for sustainable pest control strategies.

## 1. Introduction

The ability to defend against pathogens is a universal characteristic of living organisms. Unlike vertebrates, which possess an adaptive immune system, insects rely entirely on an innate immune system—comprising both humoral and cellular immunity—to combat a range of threats, from viruses and bacteria to fungi [1,2,3,4,5]. The efficiency of this system depends heavily on the recognition of pathogen-associated molecular patterns (PAMPs) by pattern recognition receptors (PRRs). Among the various defense proteins, prophenoloxidase (PPO) and C-type lectins (CTLs) have garnered significant attention due to their roles in melanization, wound healing, and the regulation of antimicrobial peptides [6,7,8,9].

Beyond their classical immune function, accumulating evidence suggests that PPOs play roles in multiple physiological processes, including wound healing, cuticle formation, development, and metabolic regulation. PPOs are synthesized as inactive zymogens and secreted by hemocytes into the hemolymph, where they are proteolytically activated into phenoloxidase (PO) through a tightly regulated serine protease cascade following immune challenge or tissue damage [10,11,12,13]. The activated PO system catalyzes the oxidation of phenolic substrates such as tyrosine into quinones, ultimately leading to melanin deposition [14,15,16,17,18]. This melanization response not only encapsulates the invading pathogen but also contributes to wound sealing and tissue repair, thereby maintaining physiological homeostasis [19].

In Lepidoptera, prophenoloxidase (proPO) is predominantly synthesized by a specialized subpopulation of hemocytes known as oenocytoids. However, lower levels of proPO have also been detected in granulocytes and plasmatocytes, as reported in the silkworm *Bombyx mori* [20]. Notably, studies in *B. mori* have revealed that proPO is capable of translocating from the hemolymph into the cuticle. Asano and Ashida (2001) demonstrated that cuticle-associated proPO exhibits exposed oxidized methionine residues on its surface, a biochemical feature that clearly distinguishes it from hemolymph-derived proPO [21,22,23,24,25,26,27]. This structural modification is thought to be associated with transepithelial transport and is likely to be the functional involvement of PPO in cuticle hardening and sclerotization. In Diptera, the cellular sources of PPOs exhibit pronounced diversification. In *Drosophila melanogaster*, PPO1 and PPO2 are produced by crystal cells, whereas lamellocytes synthesize PPO3. Following septic or aseptic injury, crystal cell-derived PPOs supply phenoloxidase activity in the hemolymph, with PPO1 being rapidly released, while PPO2 is retained within crystalline inclusions, thereby contributing to the late-stage immune response [24,25]. In other Dipteran species, such as the mosquito *Anopheles gambiae*, both oenocytoids and granulocytes can produce proPO. However, each PPO isoform exhibits a distinct expression pattern: PPO6 is broadly expressed across all hemocyte types; PPO2, PPO4, PPO5, and PPO9 are primarily expressed in granulocytes; whereas PPO1, PPO3, and PPO8 are concentrated in oenocytoids [27]. This cell-specific partitioning of PPO isoform expression reflects a detailed division of labor among hemocytes, while also underscoring the complexity and specialization inherent in insect immune regulation.

C-type lectins (CTLs) are found broadly in vertebrates, insects, and other invertebrates. Defined by at least one carbohydrate recognition domain (CRD) [28], CTLs are involved in various functions, such as sugar binding, cell adhesion, prophenoloxidase activation, and antimicrobial peptide regulation [29]. Insect CTLs are grouped into CTL-S (single CRD), IMLs (dual CRDs), and CTL-X (CRD plus other functional domains) based on their CRD count [30]. These proteins usually contain at least one C-type lectin-like domain (CTLD), which is made up of 110–130 amino acids and folds into a globular structure. This structure includes two α-helices, five β-strands, and extended loops stabilized by disulfide bonds. Shen et al. (2021) stated that CTLs play diverse roles, such as identifying and attaching to carbohydrate molecules, mediating cell–cell interactions, initiating prophenoloxidase activation, and regulating the production of antimicrobial peptides [29]. Previous research regarding C-type lectins in *D. melanogaster* highlighted that two CTL-S genes (*DL2* and *DL3*) are capable of agglutinating *Escherichia coli* in a calcium-dependent manner. These lectins were also found to bind hemocytes while promoting encapsulation and melanization processes [31].

The antimicrobial efficacy of CTLs has also been observed in the species *Chrysomya megacephala*; crude extracts and purified lectins derived from this species have been demonstrated to significantly inhibit the proliferation rate of various pathogenic microorganisms spanning both Gram-negative and Gram-positive bacteria, such as *E. coli*, *Enterococcus casseliflavus*, *Micrococcus luteus*, and *Paenalcaligenes hermetiae.* Mechanistically, the interaction between the purified lectins and these bacterial isolates triggers an increase in optical absorbance values at a wavelength of 260 nm [32]. This biophysical phenomenon indicates the leakage of intracellular material, such as nucleic acids, providing irrefutable evidence that bacterial cells undergo lysis due to disruption in the integrity of the cytoplasmic membrane caused by lectin activity. Within the order Lepidoptera, studies on *Bombyx mori* have successfully identified several CTL variants such as BmCTL-S2, -S3, and -S6 that exhibit a broad binding spectrum toward various bacterial surface ligands, including lipopolysaccharide (LPS), peptidoglycan (PGN), and other fungal cell wall components [30,33,34]. Interestingly, lectins featuring a tandem CRD structure, such as BmCTL-5, are known not only to function in pattern recognition but also to be involved in modulating the Jak/STAT signaling pathway, a major cellular communication route for responding to infection and biological stress [35].

Studies on *Manduca sexta* IML-2, an early-identified C-type lectin in lepidopteran insects, have demonstrated that it possesses the capacity to bind to an exceptionally broad spectrum of polysaccharides, including LPS, lipoteichoic acid of polysaccharides, LPS lipoteichoic acid (LTA), mannan, laminarin, and lipid A [36]. IML-2 has two CRDs, just like other immulectin group members. The tandem CRD structure is specific to the immulectin group and differs from most animal C-type lectins, which have only one CRD [37]. As a PRR, IML-2 can enhance encapsulation and melanization, phagocytosis, and proPO activation in *M. secta,* among other immune responses [38].

*Hyphantria cunea,* commonly known as the fall webworm, is a highly polyphagous invasive forest pest characterized by its exceptional adaptability and high fecundity [39]. This adaptability is supported by the fact that its larvae consume the leaves of over 400 species of deciduous trees, including those found in urban gardens [40]. This insect presents a significant danger to ecological stability and limits the potential for expansion within the Chinese agricultural and forestry economy [41]. Furthermore, to manage the *H. cunea* population, several strategies have been implemented, such as the use of natural enemies, microbial treatments, and chemical insecticides. Among these, the *H. cunea* nucleopolyhedrovirus (HcNPV) has emerged as a promising candidate because of its high host specificity and its harmlessness to beneficial organisms. Nevertheless, the practical application of this virus as a control agent is often constrained by its limited host range, delayed lethality, and inconsistent biological activity [42]. Currently, scientific efforts to utilize nucleopolyhedroviruses (NPVs) as biocontrol agents primarily revolve around several sophisticated optimization strategies. First, researchers are increasingly employing heterologous recombination techniques to engineer recombinant viruses, a process aimed at overcoming host-range limitations and enhancing the virus’s ability to target diverse pests. Second, there is a significant focus on exploring synergistic interactions by combining NPVs with *Bacillus thuringiensis*, which serves to accelerate the onset of action and overall efficacy in the field. Finally, investigators are evaluating the integration of various chemical additives and enhancers into NPV formulations to bolster their insecticidal potency and ensure more consistent results in agricultural and forestry applications [40]. Additionally, substantial research has explored the specific molecules, signaling pathways, and biological mechanisms that govern how various insect species respond to viral infection [43,44,45]. However, while these broad mechanisms are becoming clearer, the specific contributions of key immune regulators within the invasive pest *H. cunea* are still poorly understood. In particular, the roles of CTL14 and PPO4 in its developmental biology remained largely unexplored. To address this gap, the current study investigates the physiological contributions of these two genes to both larval development and antiviral immunity. The findings of this study enhance our understanding of the functional roles of CTL14 and PPO4 in *H. cunea* and provide a scientific basis for designing HcNPV synergists, paving the way for more effective and environmentally sustainable biological pest control strategies.

## 2. Materials and Methods

### 2.1. Insect Rearing

*H. cunea* eggs and artificial diets were obtained from the Ecology and Nature Conservation Institute, Chinese Academy of Forestry (Beijing, China) [46]. Larvae hatched from the egg masses were reared in an artificial climate chamber maintained at 25 ± 1 °C and 70 ± 5% relative humidity, under a 16 h:8 h (L:D) photoperiod.

### 2.2. Characteristics of Genes and Expression Profiling Analysis

The predicted nucleotide sequence of immune-related genes were obtained from published *H. cunea* transcriptome data by tBlastn in the National Center for Biotechnology Information (NCBI). Conserved domains were predicted using the website (http://www.ncbi.nlm.nih.gov/Structure/cdd/wrpsb.cgi) accessed on 15 December 2024, and defense-related genes were retrieved from GenBank using BLAST (https://blast.ncbi.nlm.nih.gov) accessed on 15 December 2024 queries. The sequence identity of the immune-related protein genes was determined with BioEdit software (v7.1.3). For phylogenetic analysis, species sequences were obtained from NCBI, and the phylogenetic tree was constructed using MEGA 6.0 based on the neighbor-joining method with a bootstrap value of 1000 replicates.

### 2.3. RNA Isolation and First-Strand cDNA Synthesis

Total RNA was extracted from eggs, 1st to 7th instar larvae, pupae, adults, and ten tissues (the head, silk glands, foregut, midgut, hindgut, epidermis, testis, ovary, Malpighian tubules, and fat body of the 1st day to 7th instar larvae) using the RNeasy Mini Kit (Qiagen, Redwood City, CA, USA) according to the manufacturer’s instructions. RNA quantity was measured using a Nanodrop ND-1000 spectrophotometer (Invitrogen, Carlsbad, CA, USA), and RNA quality was verified by 1% gel electrophoresis. RNA samples that met quality criteria were reverse-transcribed into first-strand cDNA with the Script^®^ RT Reagent Kit with gDNA Eraser (Perfect Real Time; TaKaRa, Kusatsu, Shiga, Japan) following the manufacturer’s protocol and stored at −20 °C until use.

### 2.4. RNA Inference and Antiviral Assay

The double-stranded RNA (dsRNA) was generated with the MEGAscript T7 High Yield Transcription kit (Ambion, Austin, TX, USA) according to the manufacturer’s instructions. The primers used for the in vitro transcription are listed in Table 1 and were synthesized by Sangon Biotech Co. Ltd. (Shanghai, China). The 4th-instar larvae were injected with 1 μg dsRNA (500 ng/μL) of each target gene, which was microinjected into the second-to-last segment of the abdomen using a microsyringe (Hamilton, Bonaduz, Switzerland, Hamilton #489323). ds*GFP* was used as a control dsRNA injection. Thirty larvae were injected for each treatment. Three living larvae were randomly selected at 48 h, 72 h, and 96 h post-injection to detect mRNA expression. Three biological replicates were performed for each treatment.

The 4th-instar *H. cunea* larvae, both normal and silenced, were reared on the artificial diet treated with NPV at 1 × 10^5^ OBs/mL. The mortality rate was used to evaluate antiviral activity. Three replicates were conducted for the normal and silenced treatments, and each replicate consisted of ten larvae.

### 2.5. Quantitative Reverse Transcription PCR (qRT-PCR) Analysis

The relative mRNA expression levels of *CTL14* and *PPO4* were quantified by qRT-PCR using a SYBR Green kit and an MJ Opticon^TM2^ machine (Bio-Rad, Hercules, CA, USA). Each reaction was performed in a total volume (20 μL) containing 10 μL of SYBR Green Real-time PCR Master Mix (Toyobo, Osaka, Japan), 7 μL nuclease-free water, 1 μL gene-specific primers (each; 0.5 μM), and 2 μL cDNA template (equivalent to 50 ng of total RNA). The *EF-1α* and *RPL13* genes were used as the internal reference genes. The primers were listed in Table 1. The RT-qPCR program was as follows: one cycle at 95 °C for 30 s, followed by 45 cycles at 95 °C for 12 s, 60 °C for 30 s, 72 °C for 40 s, and 82 °C for 1 s for plate reading.

The expression levels of differentially expressed genes were calculated and analyzed using the 2^−ΔΔCt^ method [47]. Each sample was repeated three times independently. The *t*-test method of SPSS 20.0 software was used to analyze the significant differences in the relative expression levels of genes between the groups (*p* < 0.05). All real-time fluorescence quantitative RT-PCR analysis results were analyzed using GraphPad Prism (v8.4.2) software.

### 2.6. Food Intake and Larval Growth

Bioassays were conducted using *H. cunea* larvae to evaluate feeding behavior and body weight changes. Fourth-instar larvae microinjected with dsRNA were reared on an artificial diet under controlled environmental conditions (16:8 h light: dark photoperiod, 25 ± 1 °C). Individual larval body weight and food consumption were measured before and after daily feeding. Data were recorded at 24, 48, and 72 h post-injection. Larval food intake and cumulative body weight gain were subsequently calculated for each treatment group. Each bioassay consisted of at least 30 larvae per treatment.

### 2.7. Reproduction Assay

Following dsRNA injection, fecundity assays were conducted by pairing gene-silenced *H. cunea* adults. After mating, the total number of eggs laid by each female was recorded, and the egg hatching rate was subsequently determined.

### 2.8. Expression and Purification Protein Analysis

After removing the signal peptide from the CTL14 sequence, PCR was carried out using specific primers (Table 1) to produce the recombinant protein. The PCR result was then cloned into a pET-28a vector. *Escherichia coli* BL21 (DE3), a chemically competent cell, was infected with the recombinant plasmid. The *E. coli* strain (Kangtai, Shenzhen, China) was then exposed to 1 mM isopropyl-β-D-thiogalactopyranoside (IPTG) for induction. *E. coli* was transformed using the original pET-28a vector as the negative control. *E. coli* protein extract buffer (HaiGene, Harbin, China) was utilized to gather the expressed protein following IPTG induction. Ni-NTA resin (HaiGene, Harbin, China) was then used to purify the 6His-tagged CTL14 and PPO4 as well as the original pET-28a. Briefly, induced *E. coli* BL21 (DE3) cells were lysed in GuNTA-0 buffer supplemented with 1 mM PMSF, and the clarified supernatant/pellet (total protein fraction) was loaded onto a pre-equilibrated Ni-NTA column. The flow-through fraction and proteins eluted with increasing concentrations of imidazole (20, 50, 100, and 250 mM) were analyzed by sodium dodecyl sulfate–polyacrylamide gel electrophoresis (SDS–PAGE), and the purified proteins were subsequently used for the following experiments. The Bradford Protein Assay Kit (Beyotime, Shanghai, China) was used to measure the concentration of the purified protein after a standard protein curve was created using bovine serum protein (BSA).

### 2.9. Antiviral Activity of CTL14 and PPO4 Protein to HcNPV

The evaluation of antiviral activity was conducted by adopting the procedure developed by Yan et al. (2024) [48]. The purified and lyophilized CTL14 and PPO4 proteins were mixed with HcNPV. The total volume of the mixture was adjusted to 1 mL using PBS buffer (137 mM NaCl, 2.7 mM KCl, 2 mM KH_2_PO_4_, and 10 mM Na_2_HPO_4_) at pH 7.4. The final concentrations of CTL14 and PPO4 proteins were set at 300 μg/mL, and the HcNPV concentration was 3 × 10^8^ OBs/mL. The mixtures containing CTL14 + HcNPV and PPO4 + HcNPV were incubated at 25 °C for 1 h before application. Then, 1 μL of the incubated mixture was microinjected into the body of *H. cunea* larvae at the 4th instar stage. As a negative control, PBS + HcNPV at a concentration of HcNPV at 3 × 10^8^ OBs/mL was used. Each treatment included three biological replicates with 30 individuals per replicate. The mortality rate of *H. cunea* larvae was monitored at regular intervals to validate antiviral efficacy and to investigate the role of target genes in the host response to HcNPV infection.

## 3. Results

### 3.1. Gene Identification and Phylogenetic Analysis

The open reading frame (ORF) of the *PPO4* gene of *H. cunea* is 2046 bp long, encoding 681 amino acids, with a predicted molecular mass of 78.3 kDa and a theoretical isoelectric point (pI) of 9.18. According to the signal peptide prediction results on the online website SignlP 5.0 Server, it shows the absence of a signal peptide. The results of multiple sequence alignment showed that the amino acid sequence identity between the *H. cunea* PPO4 and the *Bombyx mandarina* PPO4 was the highest.

**Figure 1:**
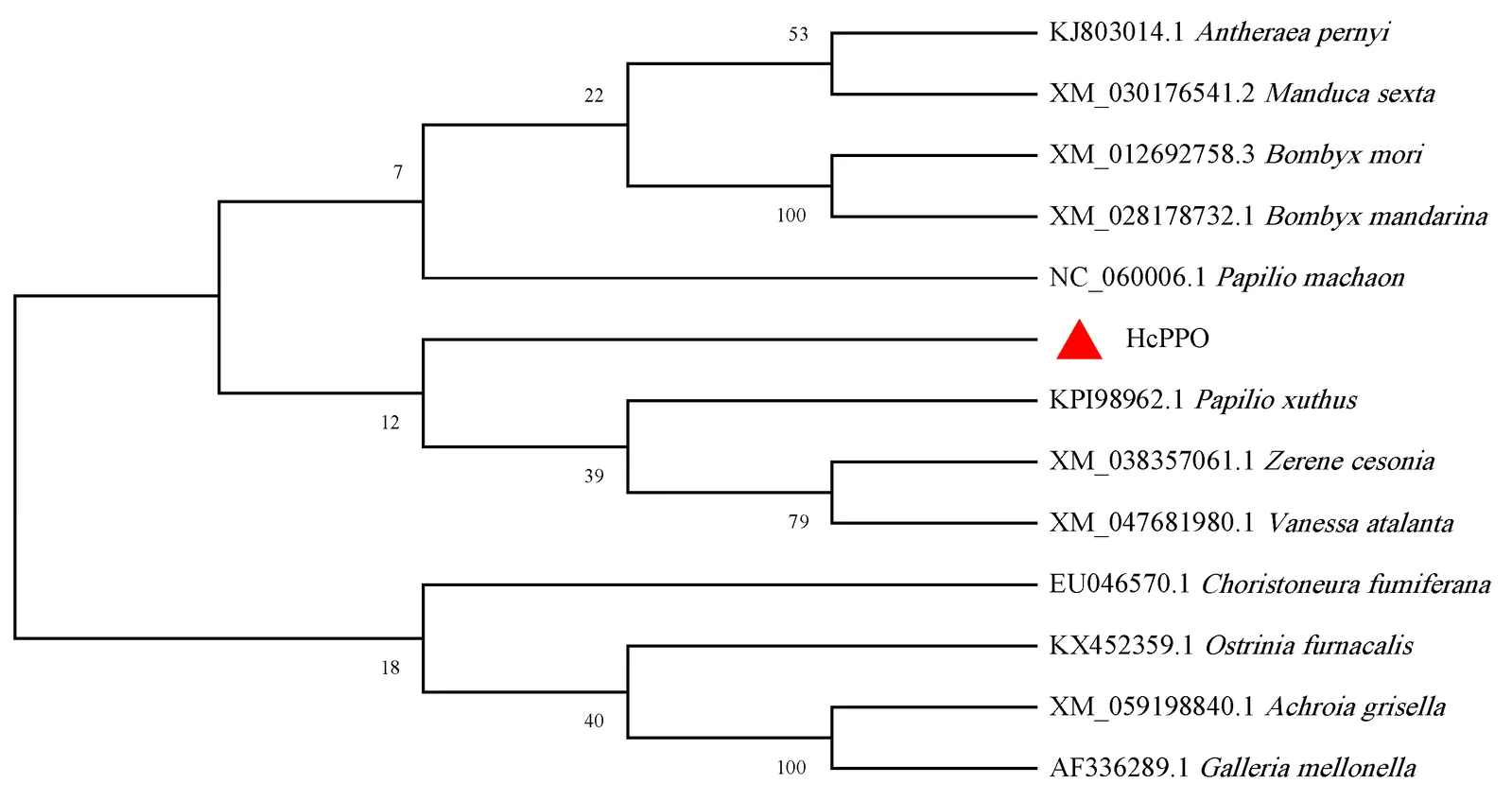
Phylogenetic analysis of PPO4 proteins.

Furthermore, to understand the phylogenetic relationships between PPO4 and other insect homologs, a 1000-bootstrap neighbor-joining phylogenetic tree was constructed as shown in Figure 1. The tree included PPO sequences from 12 representative insect species together with the PPO4 sequence of *H. cunea*. *H. cunea* PPO4 clustered with the clade comprising *Papilio xuthus*, *Zerene cesonia*, and *Vanessa atalanta*, whereas *Antheraea pernyi*, *Manduca sexta*, *Bombyx mori*, and *Bombyx mandarina* formed a separate clade. The bootstrap values at the corresponding nodes indicate the support for the inferred phylogenetic relationships.

The cDNA-encoding region *CTL14* was cloned and sequenced for confirmation. The full sequence analysis showed that. The open reading frame (ORF) of the *CTL14* gene of *H. cunea* is 1035 bp long, encoding 344 amino acids, with a theoretical pI of 5.55, and the molecular weight of the encoded protein is predicted to be 38.9 kDa. The signal peptide prediction results of the online website SignlP 5.0 Server showed that the signal peptide is 19 amino acids.

**Figure 2:**
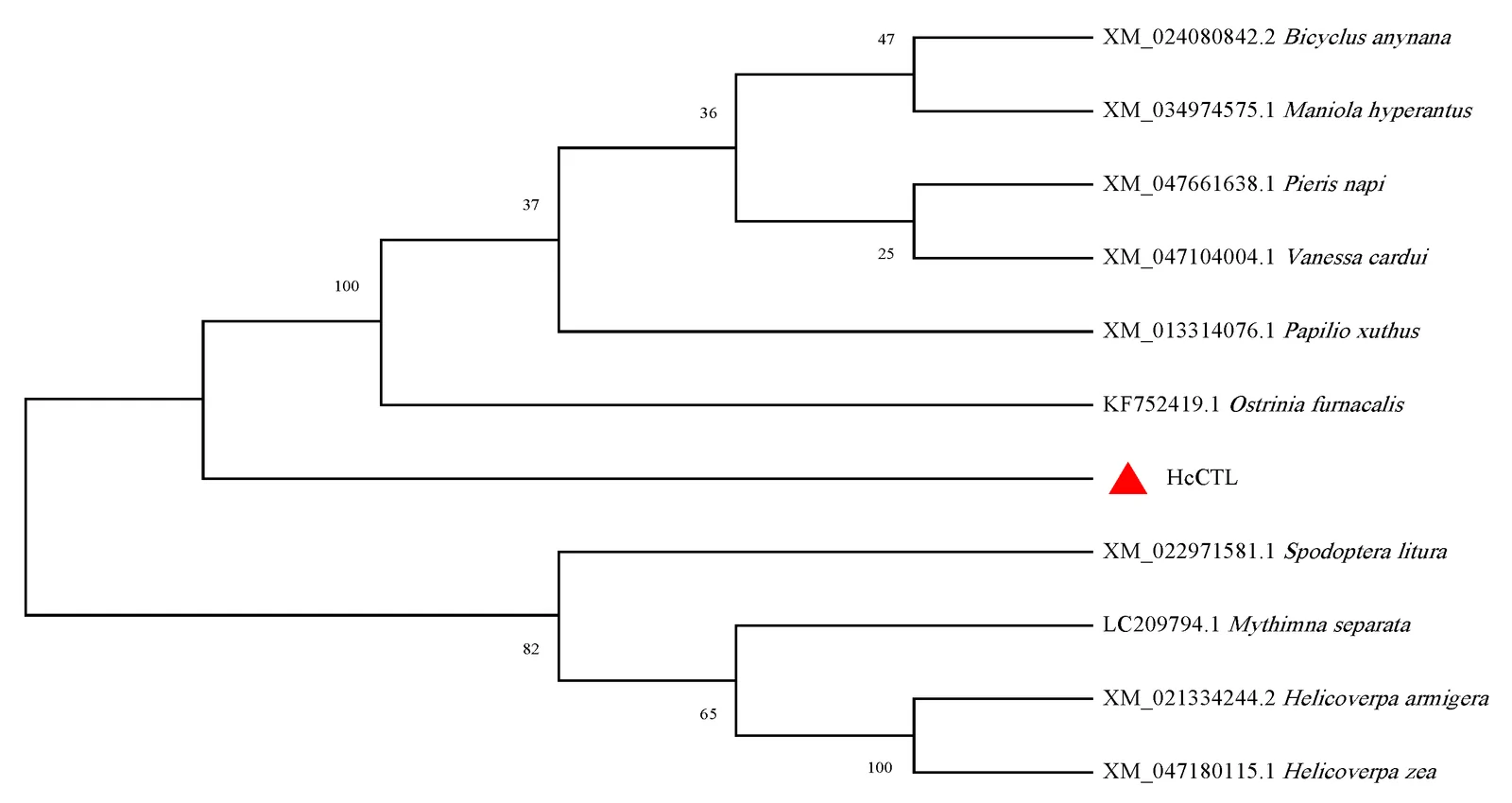
Phylogenetic analysis of CTL14 proteins.

To understand the phylogenetic relationship of CTL14, a 1000-bootstrap neighbor-joining phylogenetic tree was constructed using CTL14 to compare with other C-type lectins and lectin-related proteins from other insects. The phylogenetic tree is shown in Figure 2. There are 11 branches representing 11 different species. *H. cunea* occupies the lowest outgroup position. It separates from the large clade containing other species such as *S. litura* and *H. armigera*.

### 3.2. PPO14 and CTL14 Expressed in Different Tissues and Development

**Figure 3:**
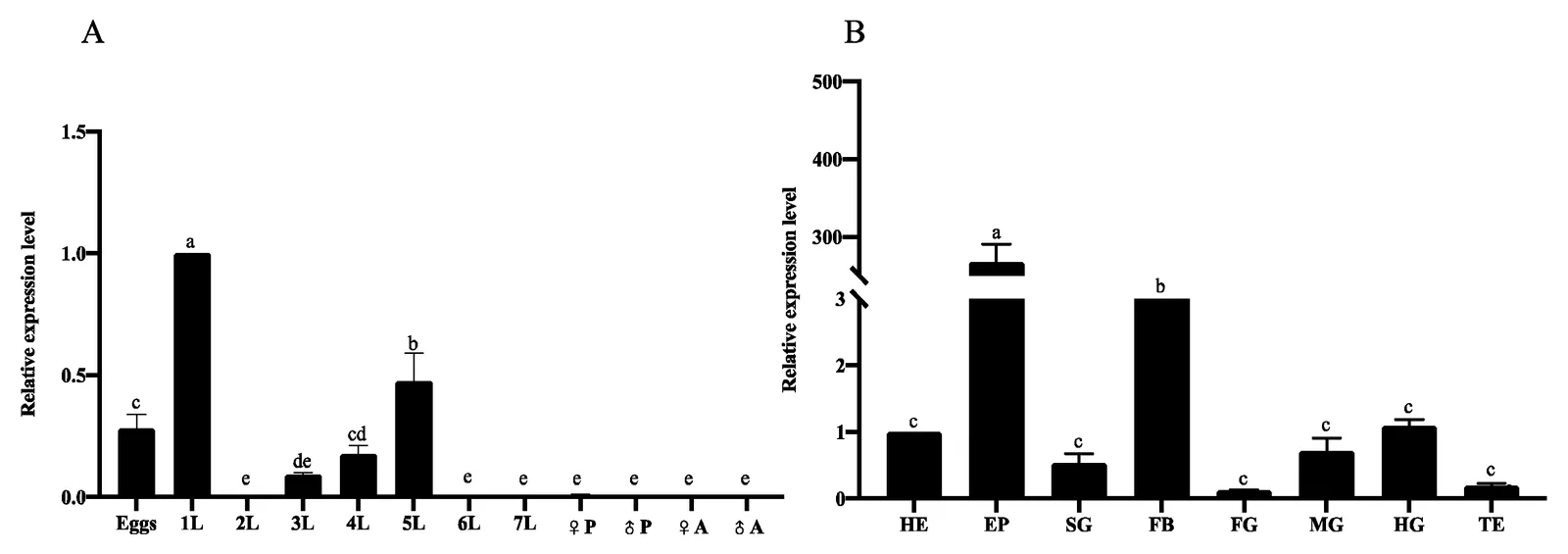
Relative expression levels of *PPO4* in different developmental stages and tissues of *H. cunea*. Eggs, first- to seventh-instar larvae, pupae, adults, and tissue samples from first-day seventh-instar larvae, including the head, epidermis, silk gland, foregut, midgut, hindgut, fat body, testis, ovary, and Malpighian tubules, were analyzed. (**A**) Expression levels across different developmental stages. Values 1L–7L represent the first- to seventh-instar *H. cunea*; ♀P, female pupa; ♂P, male pupa; ♀A, female adult; and ♂A, male adult. (**B**) Expression levels across different tissues. HE, EP, SG, FB, FG, MG, HG, TE indicate the head, epidermis, silk gland, fat body, foregut, midgut, hindgut, testis, respectively. Different letters (a–e) indicate significant differences among groups (*p* < 0.05).

The relative mRNA expression levels of *PPO4* were measured across different developmental stages and larval tissues. The results indicated that *PPO4* expression levels peaked in the first instar larvae—which served as the control—followed by the fifth instar (Figure 3A). Among the various tissues and organs, *HcPPO4* was predominantly expressed in the epidermis and fat body, reaching 267-fold and 49.5-fold higher than those in the control (head), respectively. By contrast, expression levels were lower in the testis and foregut, where transcript levels were 5-fold and 8-fold lower than the control (Figure 3B).

**Figure 4:**
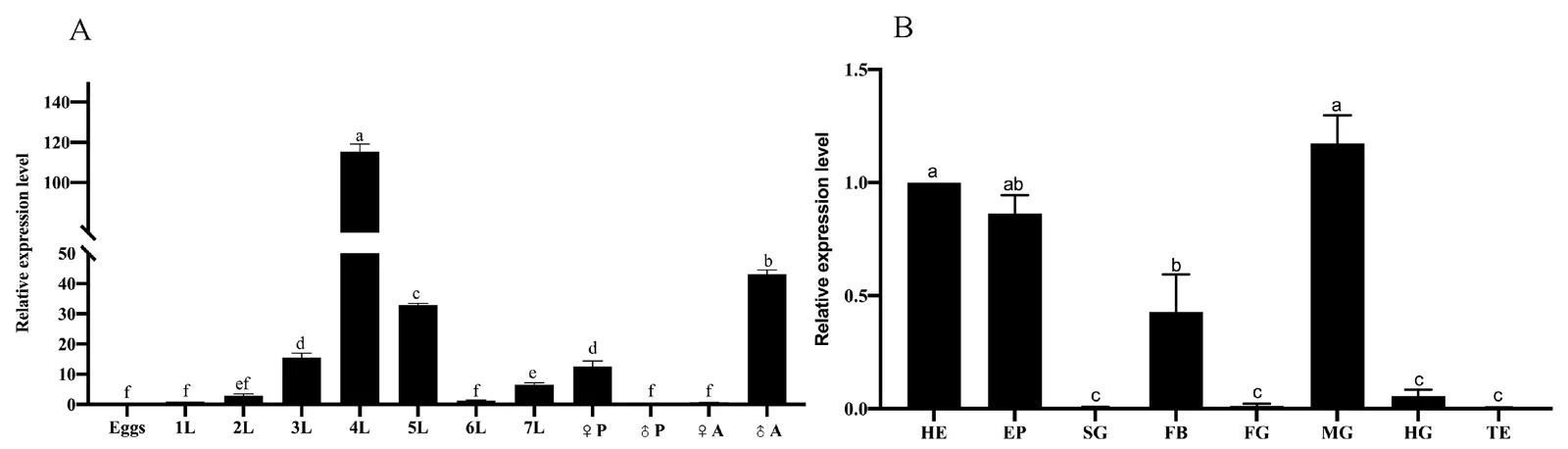
Relative expression levels of *CTL14* in different developmental stages and tissues of *H. cunea*. Eggs, first- to seventh-instar larvae, pupae, adults, and tissue samples from first-day seventh-instar larvae, including the head, epidermis, silk gland, foregut, midgut, hindgut, fat body, testis, ovary, and Malpighian tubules, were analyzed. (**A**) Expression levels across different developmental stages. Values 1L–7L represent the first- to seventh-instar *H. cunea*; ♀P, female pupa; ♂P, male pupa; ♀A, female adult; and ♂A, male adult. (**B**) Expression levels across different tissues. HE, EP, SG, FB, FG, MG, HG, TE indicate the head, epidermis, silk gland, fat body, foregut, midgut, hindgut, testis, respectively. Different letters (a–f) indicate significant differences among groups (*p* < 0.05).

The mRNA expression levels of *CTL14* were measured across various developmental stages and larval tissues. The highest expression levels were observed during the fourth instar, followed by the adult female stage (♂A) and the fifth instar, reaching 115.3-, 43.1-, and 32.8-fold levels, respectively, relative to the control (first instar). Lower expression levels were detected in the male pupa (♂P), adult female, and adult stages, reaching 16.5-fold and 1.4-fold levels relative to the control (Figure 4A). Regarding tissue distribution, *CTL14* was expressed primarily in the head, midgut, and epidermis (Figure 4B).

### 3.3. Function of PPO4 and CTL14 by RNAi

**Figure 5:**
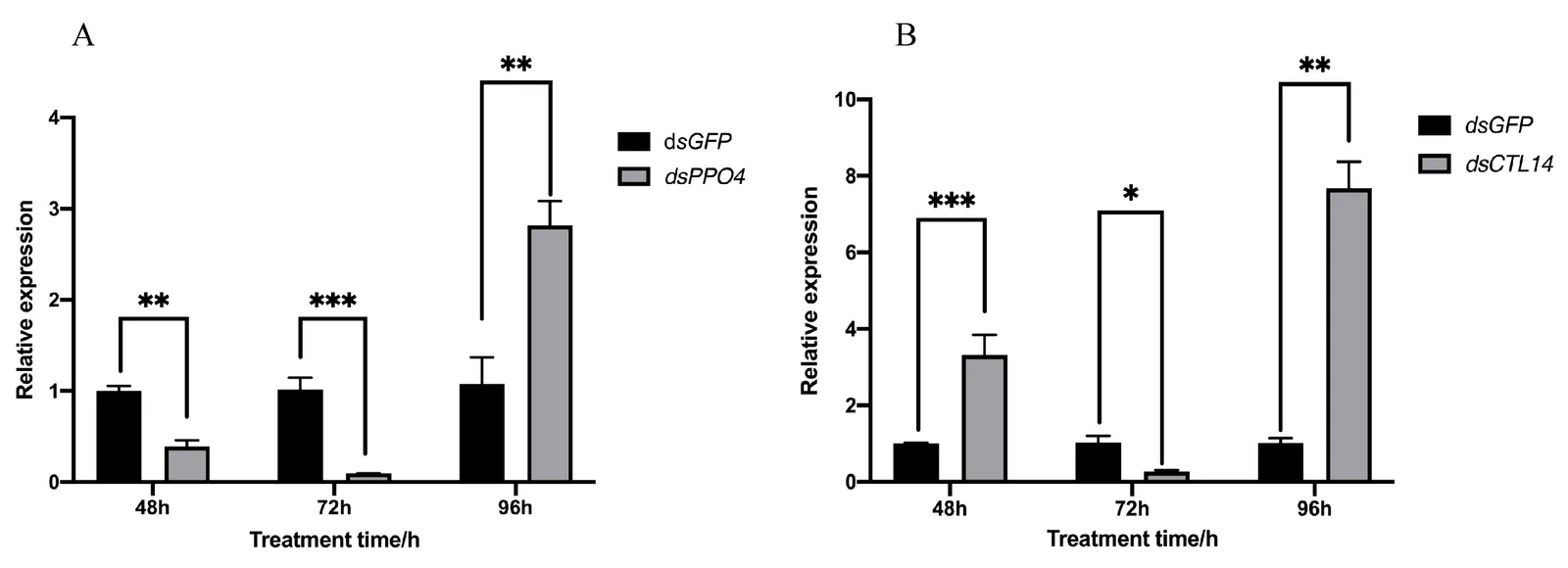
Relative gene expressions in *H. cunea* microinjected with dsRNA. (**A**) *PPO4* gene relative expression in *H. cunea* microinjected with dsRNA. (**B**) *CTL14* gene relative expression in *H. cunea* microinjected with dsRNA. Results are presented as the mean ± SEM from at least three independent experiments. Student’s *t*-test: * *p* < 0.05, ** *p* < 0.01, *** *p* < 0.001 versus ds*GFP*.

Fourth-instar *H. cunea* larvae were injected with dsRNA targeting *PPO4*, and transcript levels were subsequently quantified by qRT-PCR. Compared with the ds*GFP* control, *PPO4* expression was significantly reduced by 61% at 48 h and 91% at 72 h post-injection, indicating efficient gene silencing. However, transcript abundance markedly increased at 96 h, suggesting a recovery of *PPO4* expression following the decline of the RNAi effect (Figure 5A). Similarly, *CTL14* expression was reduced by 73.84% at 72 h, whereas transcript levels increased to 231.79% and 656.31% of the control at 48 and 96 h (Figure 5B).

**Figure 6:**
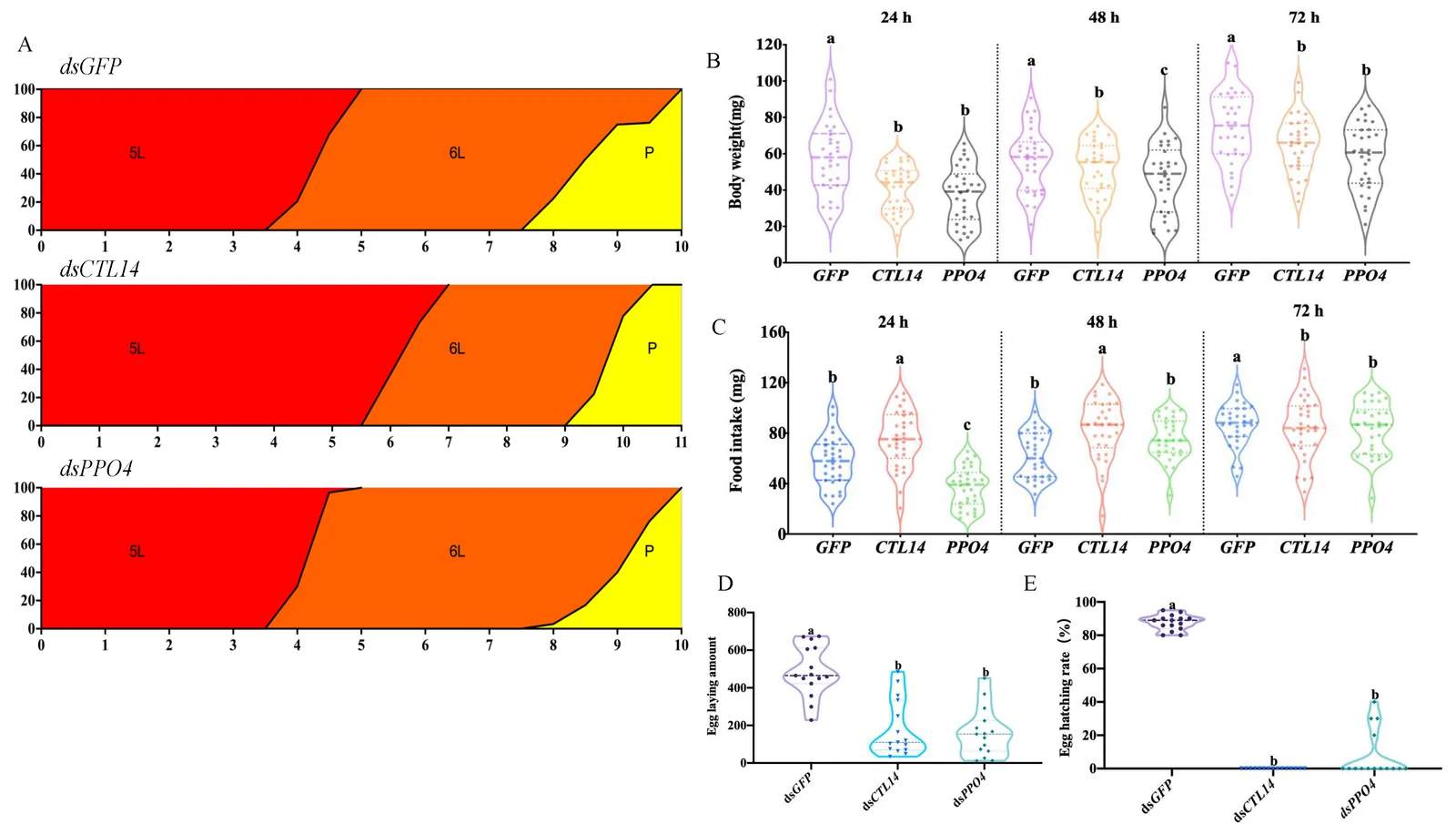
Effects of *CTL14* and *PPO4* gene silencing on the development and reproductive performance of *H. cunea*. (**A**) Developmental duration of the 5th instar to pupation. (**B**) Body weight of 5th instar *H. cunea* larvae at 24, 48, and 72 h after dsRNA injection. (**C**) Food intake of 5th instar *H. cunea* larvae at 24, 48, and 72 h after dsRNA injection. (**D**) The egg-laying amount of *H. cunea* by RNAi (E). The egg hatching rate of *H. cunea* after RNAi treatment. Different letters (a–c) indicate significant differences among groups (*p* < 0.05).

Following injection of dsRNA targeting the *PPO4* and *CTL14* genes, the developmental length of fifth-instar *H. cunea* larvae was monitored and statistically assessed (Figure 6A). As shown, fifth-instar larval developmental length was variable when the *PPO4* and *CTL14* genes were silenced. In comparison to the ds*GFP* control group, the *CTL14* dsRNA-treated group is 1.95 days longer, and the *PPO4* dsRNA-treated group is 0.85 days longer. According to these findings, silencing the *PPO4* and *CTL14* genes impacts *H. cunea* larval growth and development, prolonging the larval stage. Furthermore, dsRNA injection targeting the *CTL14* and *PPO4* genes significantly inhibited *H. cunea* larval growth, as indicated by significantly lower body weight compared to the ds*GFP* control group throughout the observation period. Within the first 24 h, the average weight of the ds*CTL14* (45–50 mg) and ds*PPO4* (~40 mg) groups already demonstrated a significant decline relative to the control (~60 mg). Up to 72 h, although all larvae experienced weight gain, the treatment groups continued to lag significantly behind, with average weight ranging only between 60 and 70 mg, while the control group surged to reach an average of 75–80 mg (Figure 6B). Regarding food intake, silencing *CTL14* and *PPO4* genes exerted a dynamic impact on the feeding activity of *H. cunea*. During the initial 24 to 48 h, ds*CTL14*-treated larvae exhibited exceptionally high feed consumption (reaching ~90 mg), surpassing the control group; conversely, ds*PPO4*-treated larvae experienced a drastic decline in appetite early on (consuming only ~40 mg) before eventually beginning to catch up. However, at the 72 h endpoint, the ds*GFP* control group ultimately demonstrated the most stable and efficient feed intake (~85–90 mg), whereas both treatment groups (ds*CTL14* and ds*PPO4*) exhibited statistically similar consumption levels (~80–85 mg) that nevertheless remained below the control level (Figure 6C).

Furthermore, the results of in-depth observations regarding the fecundity of female *H. cunea* following RNAi were assessed (Figure 6D). RNAi-mediated knockdown of *PPO4* significantly reduced the mean number of eggs laid to 160.93 ± 129.08 eggs, representing a 67.07% decrease relative to the ds*GFP* control. Similarly, *CTL14* silencing reduced fecundity to 182.73 ± 149.90 eggs per female, corresponding to a 62.61% reduction. In contrast, females in the ds*GFP* group produced an average of 488.73 ± 135.43 eggs, indicating that suppression of either *CTL14* or *PPO4* markedly impaired the reproductive capacity of *H. cunea*.

In addition, the effects of *CTL14* and *PPO4* silencing on progeny viability were evaluated by determining the egg hatching rate (Figure 6E). The average hatching rates of *H. cunea* eggs after silencing *PPO4* and *CTL14* were 8 ± 14.24% and 0.00 ± 0.00%, respectively, compared with 87.60 ± 4.64% in the ds*GFP* control group. These findings indicate that RNAi-mediated silencing of *CTL14* and *PPO4* severely impaired egg hatchability, suggesting that both genes play essential roles in embryonic development and progeny viability in *H. cunea*.

**Figure 7:**
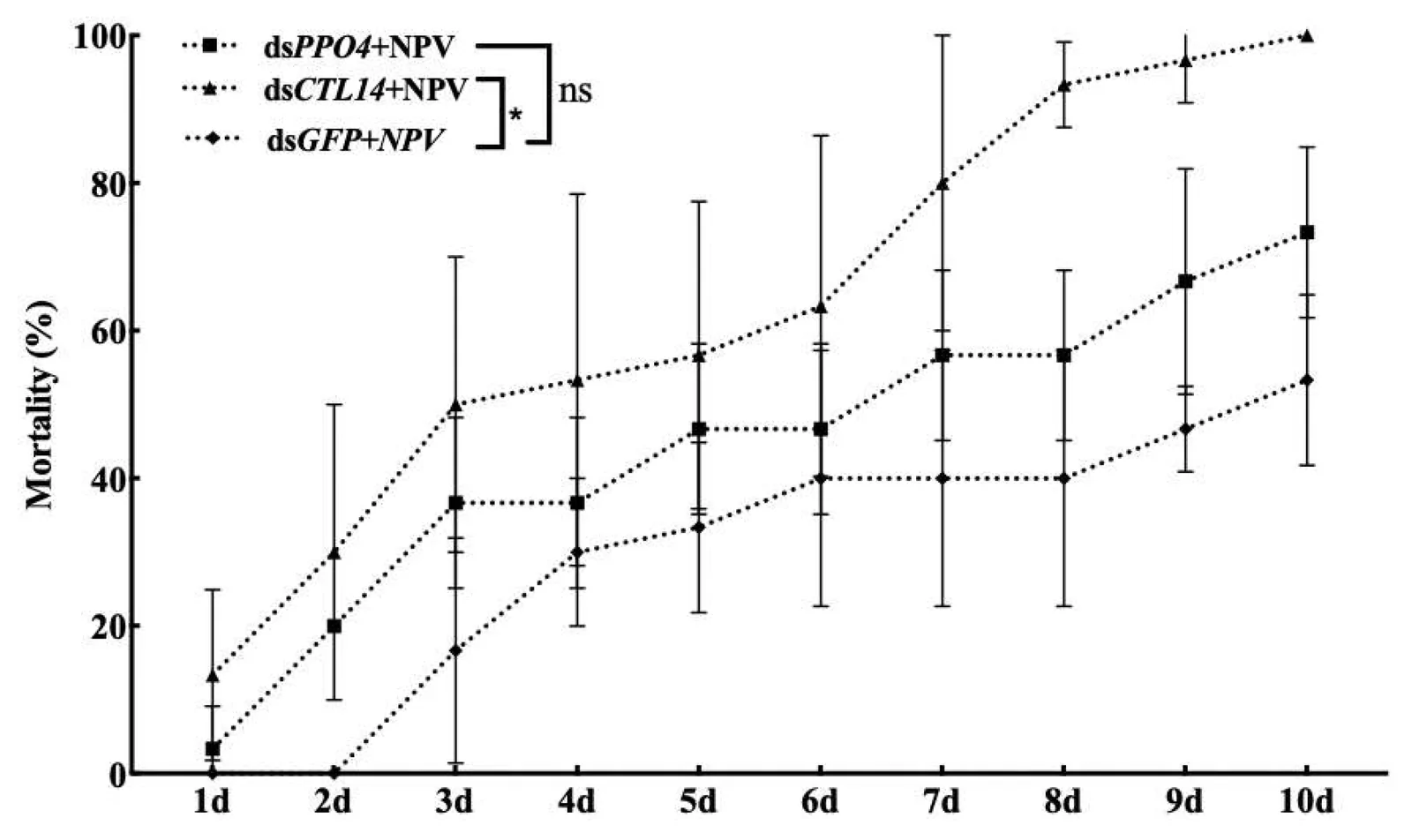
Mortality rate of fourth-instar *H. cunea* larvae after silencing *CTL14* and *PPO4* genes and exposure to HcNPV virus. Results are presented as the mean ± SEM from at least three independent experiments. Student’s *t*-test: * *p* < 0.05; ns, not significant.

The effects of *CTL14* and *PPO4* silencing on antiviral immunity were further evaluated by monitoring the mortality rate of fourth-instar *H. cunea* larvae following HcNPV challenge (Figure 7). Larvae treated with ds*CTL14* or ds*PPO4* exhibited significantly higher mortality than those in the ds*GFP* control group, indicating that knockdown of either gene increased susceptibility to HcNPV infection. The ds*CTL14* group showed the most rapid disease progression, with mortality reaching approximately 50% by day 3 and 100% by day 10. Similarly, mortality in the ds*PPO4* group increased steadily, reaching approximately 75% by the end of the experiment. In contrast, mortality in the ds*GFP* control group increased more gradually, reaching approximately 40% by day 7 and stabilizing at 50–55% by day 10, indicating greater resistance to viral infection.

### 3.4. Recombinant Expression and Purification

**Figure 8:**
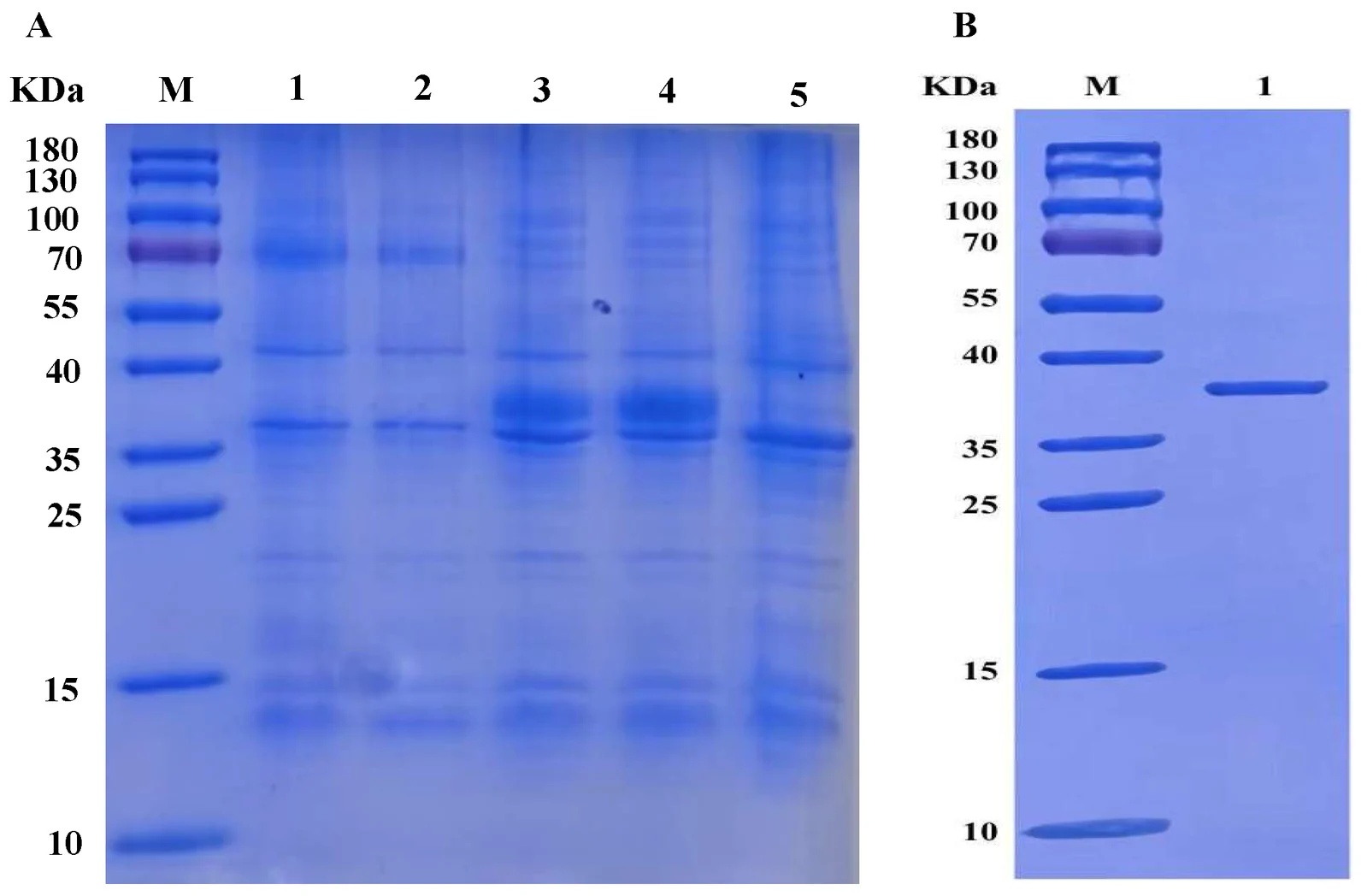
SDS-PAGE analysis of the recombinant CTL14 protein. (**A**) SDS-PAGE analysis of the recombinant CTL14 protein expression in *E. coli* induced by IPTG. M, protein marker; Lane 1, 1 mM IPTG, 16 h at 37 °C; Lane 2, 0.6 mM IPTG, 16 h at 37 °C; Lane 3, 1 mM IPTG, 16 h at 16 °C; Lane 4, 0.6 mM IPTG, 16 h at 16 °C; Lane 5, non-induced. (**B**) Purification of recombinant protein in *E. coli* cell lysates. M, protein marker; Lane 1, purified recombinant protein.

**Figure 9:**
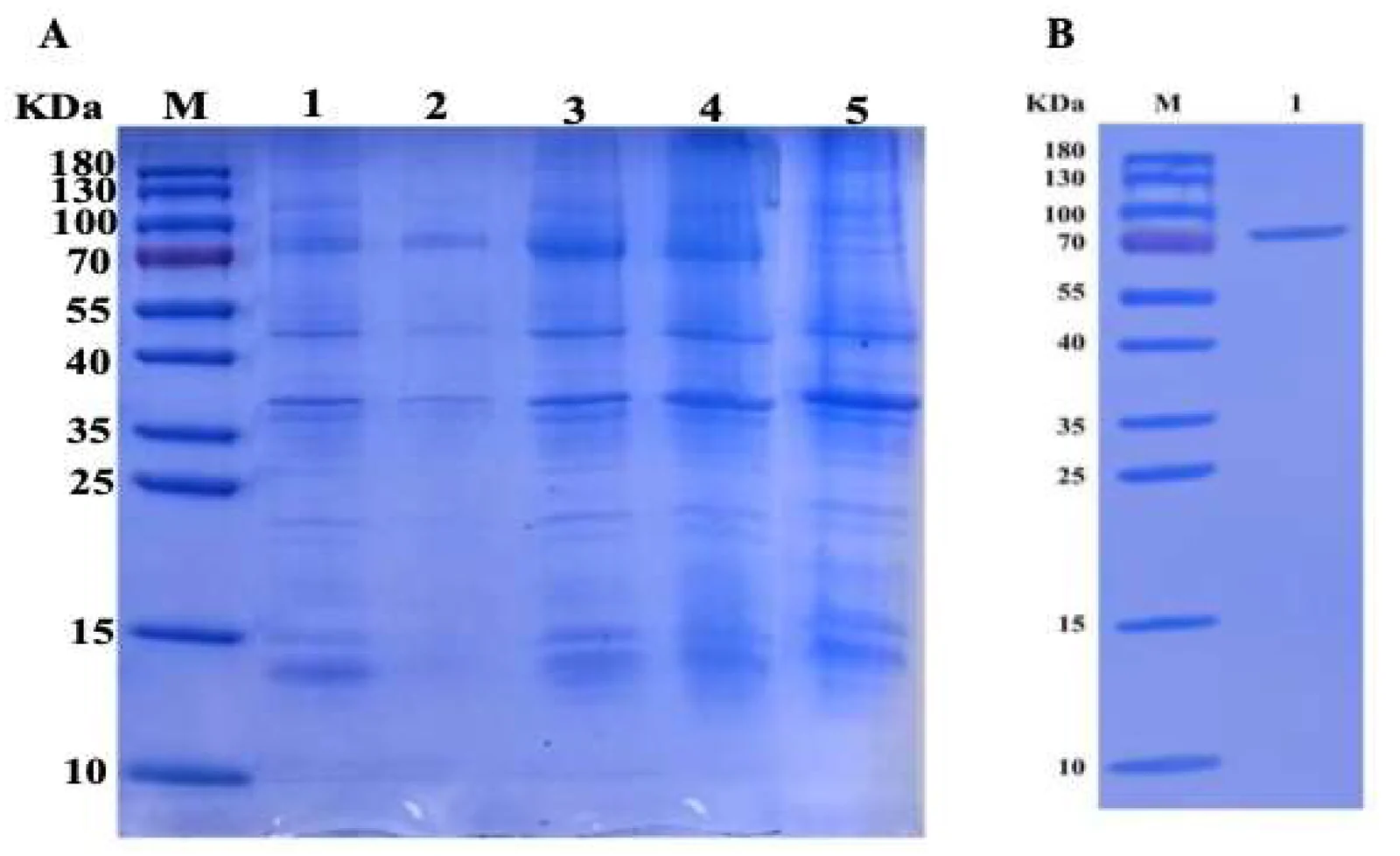
SDS-PAGE analysis of the recombinant PPO4 protein. (**A**) SDS-PAGE analysis of the recombinant PPO4 protein expression in *E. coli* induced by IPTG. M, protein marker; Lane 1, 1 mM IPTG, 16 h at 37 °C; Lane 2, 0.6 mM IPTG, 16 h at 37 °C; Lane 3, 1 mM IPTG, 16 h at 16 °C; Lane 4, 0.6 mM IPTG, 16 h at 16 °C; Lane 5, non-induced. (**B**) Purification of recombinant protein in *E. coli* cell lysates. M, protein marker; Lane 1, purified recombinant protein.

The validated recombinant plasmid pET-28a (+)-CTL14 was transformed into *E. coli* BL21 (DE3) host cells. Cell culture was performed in liquid LB medium containing kanamycin at 37 °C with 180 rpm agitation until an OD600 of 0.6 was reached. Protein expression was induced using various concentrations of IPTG (0.6 and 1.0 mM) at 16 °C and 37 °C for 16 h. Recombinant CTL14 was successfully expressed in *E. coli* BL21 (DE3) following induction with 0.6 mM IPTG at 16 °C for 16 h. SDS–PAGE analysis revealed a prominent protein band at approximately 38.9 kDa, which was consistent with the predicted molecular weight of CTL14 (Figure 8A). The recombinant protein was subsequently purified and yielded a single protein band, indicating high purity suitable for subsequent functional analyses (Figure 8B, Supplementary Figures S1–S3). Similarly, recombinant PPO4 was successfully expressed under the same induction conditions. SDS–PAGE analysis showed a distinct protein band at approximately 78.3 kDa, corresponding to the predicted molecular weight of PPO4, whereas no corresponding band was detected in the non-induced control (Figure 9A). Following this, the purified PPO4 protein appeared as a single target band, confirming the successful purification of the recombinant protein for subsequent experiments (Figure 9B, Supplementary Figures S1–S3).

**Figure 10:**
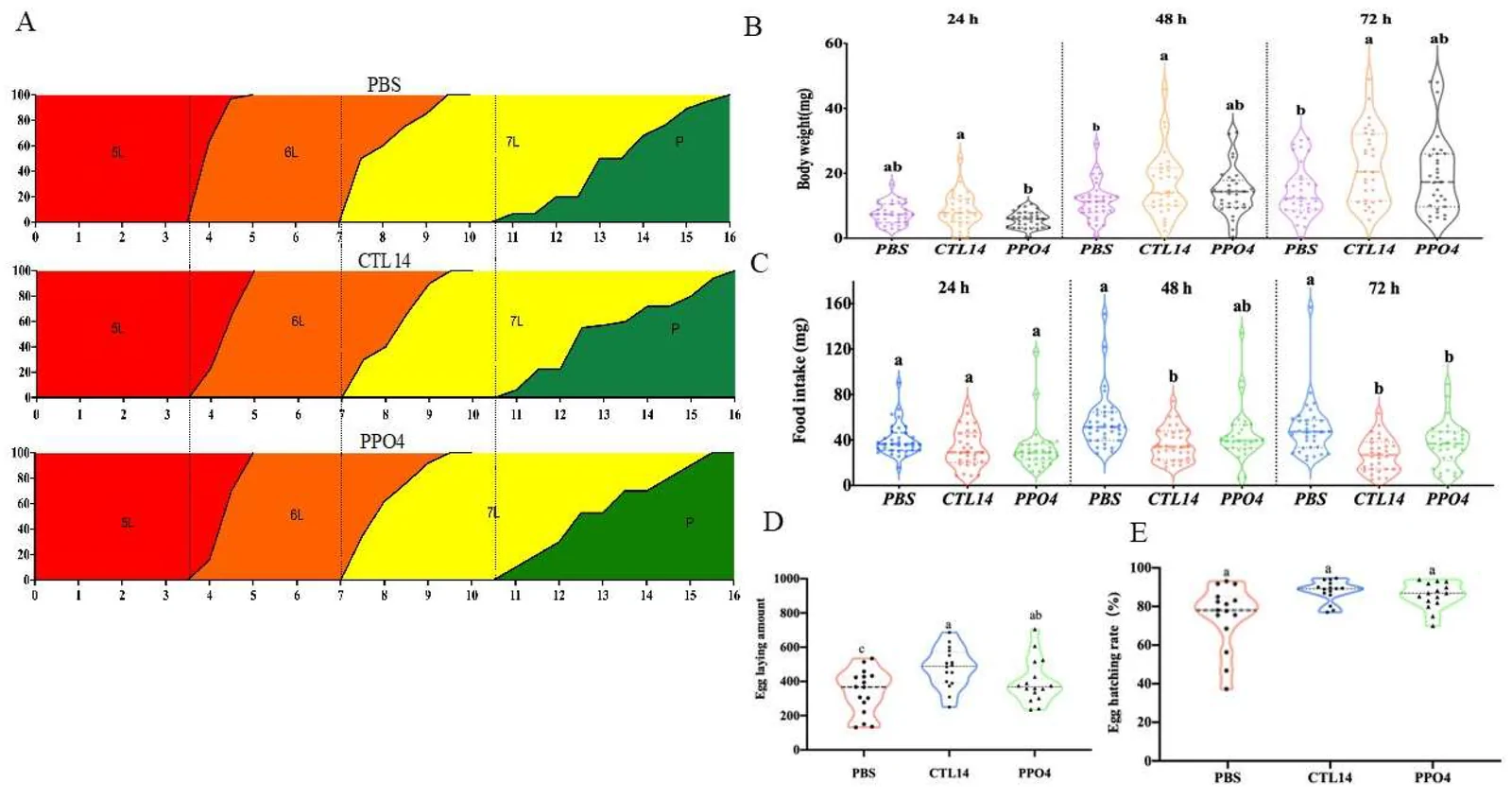
Effects of recombinant CTL14 and PPO4 protein on the development and reproductive performance of *H. cunea*. (**A**) Larval developmental time after target protein injection. (**B**) Larval body weight after target protein injection. (**C**) Larval food intake after target protein injection. (**D**) *H. cunea* egg laying after target protein injection. (**E**) *H. cunea* egg hatching after target protein injection. Different letters (a–c) indicate significant differences among groups (*p* < 0.05).

The effect of recombinant CTL14 and PPO4 proteins on the developmental duration of fifth-instar *H. cunea* larvae was evaluated using PBS-treated larvae as the control (Figure 10A). No significant differences in developmental duration were observed among the treatment groups. Larvae remained in the fifth instar from day 0 to day 3, molted to the sixth instar between days 3 and 4, and predominantly remained in the sixth instar until day 7. The transition to the seventh instar began on day 7, with most larvae remaining at this stage until day 11, while a small proportion had entered the prepupal or pupal stage by day 10.

The effects of recombinant CTL14 and PPO4 proteins on larval body weight and food intake were further evaluated following protein injection (Figure 10B,C). At 24 h post-injection, larval body weight did not differ significantly among the treatment groups. However, significant differences became evident at 48 h, with larvae treated with recombinant CTL14 exhibiting a significantly greater mean body weight (16.96 ± 10.14 mg) than those in the PBS control group (11.53 ± 6.84 mg). This trend persisted at 72 h, when the CTL14-treated larvae reached an average body weight of (23.63 ± 11.23 mg), whereas the PPO4-treated group showed intermediate values between the CTL14 and PBS treatments. In contrast, food intake was similar among all groups at 24 h after injection. At 48 h, larvae injected with recombinant CTL14 consumed significantly less food (36.03 ± 14.85 mg) than the PBS-treated larvae (55.19 ± 22.18 mg), and this difference remained significant at 72 h. The PPO4-treated group exhibited an intermediate feeding response throughout the observation period.

This study also examined the effect of protein injection on egg-laying capacity and hatchability (Figure 10D,E). The results showed that the CTL14 group exhibited a markedly higher egg-laying rate (479 ± 117.36 eggs) than both the control group (340.25 ± 129.48 eggs) and the PPO4 group (400 ± 129.32 eggs). Meanwhile, the percentage of egg hatching remained consistently above 80% across all three groups (PBS, CTL14, and PPO4). The average egg hatching rates were 79.44 ± 17.17% for the PBS control, 87.88 ± 5.53% for the CTL14 group, and 85.65 ± 6.87% for the PPO4 group, respectively.

**Figure 11:**
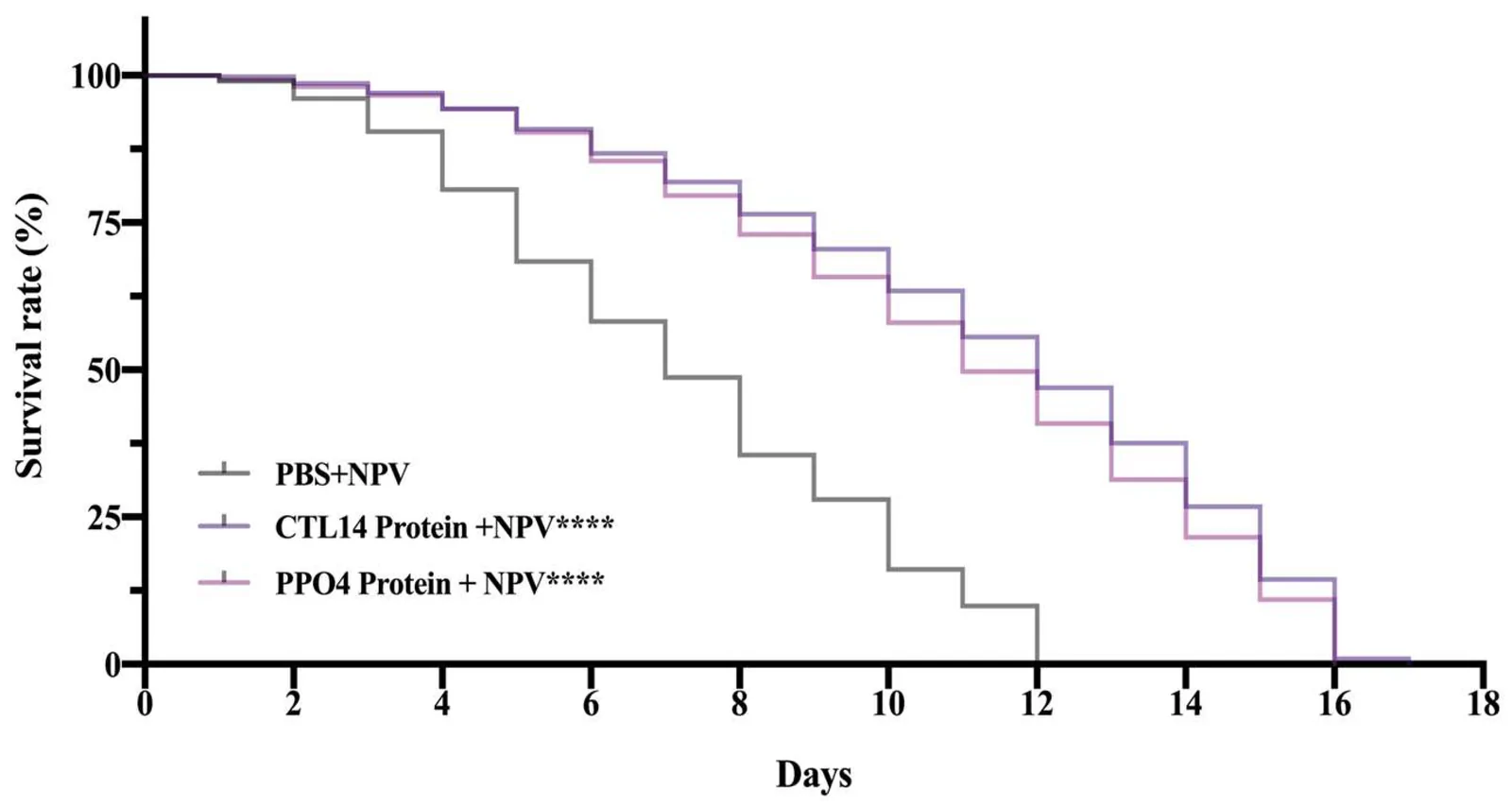
Mortality rate of *H. cunea* larvae following injection with recombinant CTL14 and PPO4. Data are presented in survival curves as assessed by the log-rank (Mantel–Cox) test. The asterisks (****) indicate significant differences at the *p* < 0.0001 level.

To evaluate the roles of these genes in mediating immune responses, *H. cunea* larvae were subjected to recombinant protein injection combined with HcNPV challenge. Our results demonstrated that the larvae co-treated with CTL14 + NPV and those receiving PPO4 + NPV exhibited significantly different mortality profiles relative to the control group (PBS + NPV). Larvae that only received PBS + NPV injection showed the steepest, fastest decline in survival. In this control cohort, viral infection symptoms intensified continuously, leading to a cumulative mortality of 100% (0% survival) by day 12 post-injection. While viral proliferation still caused larval death in the treatment groups, mortality progressed far more slowly in the CTL14 protein + HcNPV and PPO4 protein + HcNPV cohorts. Notably, survival rates remained above 0% in both treatment groups throughout the entire 16-day observation period (Figure 11).

## 4. Discussion

For insects to survive in challenging environments, innate immune responses are crucial. Pattern recognition receptors (PRRs) play a central role in the innate immune response [49]; CTLs can identify glycans from a variety of sources [36]. In arthropods, CTLs are categorized as pleiotropic proteins that play diverse roles in the innate immune system. In *Drosophila*, the C-type lectins DL2 and DL3 have been reported to enhance cellular immunity by directly interacting with hemocytes and promoting encapsulation [31]. Furthermore, the CLSP2 lectin domain in mosquitoes has been shown to aid in the agglutination of the fungus *B. bassiana* in vitro and support the defense response against this fungal infection [50]. On the other hand, the melanization mechanism plays a vital role in insect innate immunity when facing microbial attacks [14]. Melanin production during the insect immune response relies on the activation of phenoloxidase (PO), the key enzyme that catalyzes the melanization cascade [51]. This enzyme is initially produced in an inactive zymogen form, namely prophenoloxidase (PPO), whose activation is tightly controlled by a serine protease cascade (cSP).

In this study, we investigated the role of PPO4 and CTL14 in *H. cunea* in response to NPV infection. These results showed that the *CTL14* and *PPO4* genes were successfully cloned in *E. coli* using TA cloning. Additionally, expression patterns of *CTL14* immunity-related genes in different developmental stages and tissues of *H. cunea* were observed; these genes are expressed throughout the developmental process. Interestingly, the mRNA expression of *CTL14* was highly detected in the fourth instar, fifth instar, and ♂adult. CTL14 is extensively expressed in the midgut, followed by the epidermal tissues (Figure 4). Similar results were also found: *TcCTL12* mRNA was expressed at all development stages, especially highly expressed in late pupa and early adult stages of *T. castaneum* [52]. However, the majority of *Armigeres subalbatus*’s *AsCTLMA15*, *AsCTLGA5*, *AsCTL15*, and *AsCTLMA11* expression was found in hemolymph [49]. In contrast, the expression of *CTL1* and *CTL2* in *Musca domestica* was reported to reach its highest level during the pupal stage [53]. Tissue-specific expression analysis revealed that *Aalb_CTL1* transcripts accumulated predominantly in the salivary glands of female mosquitoes, with substantially lower expression in the fat body and midgut [54]. This suggests a temporal shift in immune system utilization, tailored to the specific immune strategies of each species. The findings indicate that *CTL14* may be significant in the metamorphosis of *H. cunea*.

Furthermore, we examined more detailed expression patterns of *PPO4* immunity-related genes in different developmental stages and tissues of *H. cunea* (Figure 3). The results revealed that it was higher in the first- and fifth-instar larval stages than in larvae at other stages. Among various tissues and organs, *PPO4* was primarily expressed in the epidermis and fat body. The *PPO* genes in *H. cunea* showed stage-specific developmental expression patterns, which are also observed in *Drosophila* [55]. Similarly, *PPO* genes have been reported to exhibit high expression during the larval stage of the red flour beetle, suggesting an important role in larval physiology and immune function [56]. In addition, another study on *Tribolium castaneum*’s prophenoloxidase genes and antimicrobial host immune system revealed that PPO mRNAs are comparatively prevalent in both prepupae and middle-stage pupae [57].

The functional analysis of the immune system revealed that CTL14 and PPO4 contribute significantly to developmental time, including hatching rate, mortality, and egg laying. RNA interference (RNAi) has emerged as a highly effective technique for investigating gene functionality across a diverse array of insect species [58]. Hence, RNAi was performed to interfere with *CTL14* to verify the speculation. The results showed that the relative gene expression in *H. cunea* microinjected with dsRNA was effective at 72 h after injection for *CTL14*. For the *PPO4* gene, gene silencing was effective at 48 and 72 h after injection (Figure 5). These results indicate that dsRNA injection reduced the expression and confirmed that the target genes were successfully silenced. Compared with the control group, larvae subjected to RNAi exhibited significantly lower body weight and reduced food intake. These findings indicate that silencing *CTL14* and *PPO4* adversely affected larval growth and feeding performance in *H. cunea*. The prolonged developmental duration observed after *CTL14* and *PPO4* silencing suggests that these genes contribute to normal larval development in addition to their immune functions. Similar developmental delays have been reported following RNAi-mediated knockdown of immune- and hormone-related genes in other insects, including the C-type lectin *TcCTL13* in *Tribolium castaneum*, where gene silencing impaired metamorphosis, and in lepidopteran pests in which RNAi disrupted larval growth and prolonged developmental duration. These findings support the pleiotropic roles of immune-associated genes in coordinating developmental processes together with innate immune responses [59]. Furthermore, CTL-mediated hemocyte encapsulation is an important component of insect cellular immunity, and disruption of this process has been associated with reduced larval growth and overall fitness [31]. The observation that *CTL14*-silenced larvae exhibit increased food intake during 24–48 h post-injection (Figure 6C) yet show significantly reduced body weight gain relative to control larvae (Figure 6B). The reduction in larval fitness observed in the present study is consistent with the energetic trade-off theory, whereby activation or disruption of immune responses leads to the reallocation of energy away from growth and reproduction to maintain physiological homeostasis [60]. When the immune system is damaged or stressed, such as when genes are silenced, insects have to reallocate energy. When larvae are under a lot of stress, they will give up physical growth, like weight and size, to keep their bodies in balance, or homeostasis. Silencing of *CTL14* likely disturbs immune homeostasis, triggering a compensatory metabolic response. Under such immune stress, larvae may increase food consumption in an attempt to meet elevated energetic demands; however, the assimilated nutrients are preferentially diverted to stress mitigation, immune compensation, cellular repair, and detoxification processes rather than somatic growth. Consequently, increased feeding does not translate into proportional weight gain.

Moreover, RNAi technology not only exerts lethal stress on the larval stage but also has long-term detrimental effects that extend into the adult stage of the insect. One of the most significant findings in this study was a drastic reduction in reproductive ability (fecundity) and egg hatchability (fertility) after silencing specific immune genes (Figure 6D,E). RNA interference induced in the larval stage apparently had a lasting impact until the insects reached the imago (adult) stage. Observations regarding the effects of injecting *PPO4* and *CTL14* dsRNA revealed that the number of eggs laid by adult insects decreased significantly compared to the control group. The most significant impact was found in the group treated with *CTL14* gene silencing. The ds*CTL14* group had a 0% hatching rate, which means that none of the eggs turned into larvae. This total failure indicated that the *CTL14* gene is essential for the immune system and for protecting embryos or for physiological processes that happen during embryogenesis. This discovery regarding the essential function of the *CTL* gene is corroborated by research conducted on other insect species, including *Helicoverpa armigera* [54]. These findings demonstrate that RNA interference directed at *PPO4* and *CTL14* significantly influences the physiology, growth, survival, and reproduction of *H. cunea*. Additionally, successfully silencing these genes makes their biological activities noticeable, yet it is also suggested that they could be valuable as molecular targets for pest control tactics. RNAi-based strategies may provide a promising and environmentally friendly alternative strategy for controlling the population of this important pest.

The functional validation of immune-related genes as determinants of antiviral resistance was confirmed through complementary experimental approaches combining loss-of-function and gain-of-function strategies. The injection of dsRNA targeting *PPO4* and the *CTL14* injection followed by exposure to NPV resulted in a significantly faster death rate and higher mortality compared to the control group. This occurs because silencing of the key immune genes *PPO4* and *CTL14* eliminates a primary line of the immune system just before or during a viral attack. Silencing of the first line of the immune system, the *PPO* gene, is a vital component of the melanization pathway, which serves as one of the insect’s first lines of immune defense. The activation of the body’s immune mechanism via this genetic pathway is underscored by findings demonstrating that molecular interventions, specifically the inactivation (silencing) or downregulation (knockdown) of the genes encoding these proteins using RNAi techniques, result in a surge in bacterial populations within the hemolymph and a significant reduction in mosquito survival rate. This phenomenon provides strong evidence that these proteins serve as essential components in orchestrating the host’s antimicrobial response [61]. Specifically, in the host’s *Aedes albopictus,* a C-type lectin exhibiting binding affinity for mannose, designated Aalb-CTL, has been proven to play a crucial role in suppressing infections caused by yeasts and Gram-positive (G+) bacteria [62]. However, the functional dynamics of C-type lectins reveal intriguing variability depending on the specific type of pathogen involved. In *Aedes aegypti,* the galactose-specific C-type lectin 1 (mosGCTL-1) actually acts as a facilitator, aiding the adhesion of West Nile Virus (WNV) to host cell membranes. Consistent with this observation, mosGCTL-3 has also been identified as a key factor mediating the efficiency of infection by Dengue virus serotype 2 (DENV2), indicating that viruses can exploit certain types of lectins to serve as cellular entry points [63]. In-depth investigations using modern omics, transcriptomics, and proteomics approaches have revealed the complex dynamics between pathogenic viruses and the insect *H. armigera*, demonstrating that NPV infection triggers a pronounced suppression of specific immune gene expression, namely *HaCTL5* and *HaCTL7* [64]. This downregulation indicates a viral immune subversion strategy, wherein the pathogen attempts to cripple host defense components to facilitate more effective viral replication and dissemination within the insect’s tissues.

Additionally, the purified proteins were injected into fourth-, fifth-, and seventh-instar *H. cunea* larvae, and observations were made on various fitness parameters. Developmental analysis results showed that it did not significantly alter the duration of growth; the average transition between instars remained stable at 3.5 days. This phenomenon indicates that efforts to strengthen the immune system through exogenous pathways did not disrupt the hormonal rhythms that regulate the molting cycle. Interestingly, although developmental duration remained stable, larval weight data (as presented in Figure 10) showed that the group receiving *CTL14* had significantly higher body mass at 48 and 72 h post-injection compared to the control group. This finding led to the conclusion that the protein plays a role in the same time period, which is highly superior over the same time period, which is a highly superior indicator of biological fitness [60]. On the other hand, an interesting observation was made regarding the dynamics of feed consumption, where the treatment group showed a decrease in food intake compared to the control. Based on in-depth observations, this decrease in intake occurred simultaneously with the transition phase from the 4th instar to 5th instar larvae. Therefore, this decrease is most likely a manifestation of natural pre-ecdysis (pre-molting) behavior, in which larvae tend to cease feeding before shedding their old exoskeleton. Injection of certain immune proteins has been known in some cases to accelerate the achievement of the critical body weight threshold required to trigger this molting process. Furthermore, supporting literature indicates that the decrease in food intake following immune protein injection is often accompanied by stable or even increased body weight gain, reflecting a significant surge in metabolic efficiency [65]. Physiologically, *H. cunea* larvae under normal conditions must allocate significant metabolic energy to synthesize immune proteins independently from nutrient proteins; this internal biosynthetic burden is drastically reduced. This allows larvae to consume less food while still achieving optimal body mass gain, as energy that would otherwise be allocated to immune biosynthesis pathways can be fully diverted to somatic tissue development and body growth [52,66]. Therefore, it can be assumed that the decreased food intake in larvae injected with *CTL14* and *PPO4* is not an indication of toxicity but rather reflects a higher nutrient conversion efficiently allocated to growth after the burden of immune protein synthesis was reduced by the injection of purified protein.

The antiviral activities of recombinant CTL14 and PPO4 proteins against HcNPV infection provide compelling evidence that exogenous protein supplementation can significantly enhance larval survival. Survival assays demonstrated that larvae treated with CTL14 + NPV or PPO4 + NPV exhibited markedly higher survival rates than those in the control group (PBS + NPV) (Figure 11), indicating the involvement of CTL14 and PPO4 in the antiviral immune response of *H. cunea*. At the molecular level, CTL14 functions as an early immune surveillance and physical barrier against NPV particles. As a pattern recognition receptor (PRR) [67], CTL14 exhibits high affinity for carbohydrate moieties on viral envelope glycoproteins that are essential for membrane fusion. This interaction generates steric hindrance that limits viral attachment to host cell receptors, thereby suppressing the initial stages of viral entry. In addition, CTL14 can coat viral particles and act as an opsonin, facilitating their recognition and clearance by hemocytes through phagocytosis. PPO4, in contrast, plays a pivotal role in the melanization cascade [68,69], one of the most potent humoral immune defenses in insect hemolymph. Upon pathogen recognition, prophenoloxidase is activated to phenoloxidase (PO), triggering a cascade of oxidative reactions that generate quinones and reactive oxygen species (ROS) with strong virucidal activity. These reactive intermediates can directly compromise the structural integrity of viral particles. The process culminates in the deposition of melanin around the pathogen, forming a dense capsule that restricts viral dissemination and prevents systemic spread to critical tissues, such as the fat body, the primary site of NPV replication. By limiting viral load in the hemolymph, PPO4-mediated melanization helps preserve tissue integrity and supports normal metabolic function during infection [70]. Notably, emerging evidence indicates that immune effector enzymes often exert pleiotropic functions beyond direct pathogen defense. For example, studies on the heme peroxidase HPX15 in *Anopheles stephensi* demonstrated that silencing this immune enzyme not only altered midgut bacterial homeostasis but also activated alternative immune pathways, including JAK/STAT–NOS signaling, revealing a tight coupling between immune regulation and physiological balance in insects [71,72]. Consistent with these findings, our results show that disruption of *CTL14* and *PPO4* in *H. cunea* affects not only antiviral immunity but also larval growth and development. Taken together, these findings suggest that CTLs and PPO function beyond their canonical roles in innate immunity by coordinating both antiviral and physiological processes. Disruptions of these related immune genes may alter immune-physiological homeostasis and energy allocation, thereby affecting host fitness and susceptibility to viral infection. Mechanistically, CTL14 appears to participate in viral recognition and restrict the early stages of viral entry, whereas PPO4 facilitates pathogen elimination through melanization and limits viral dissemination within the host. The complementary functions of these proteins emphasize their coordinated contribution to antiviral immunity and highlight their potential as promising molecular targets for the development of environmentally sustainable pest management strategies.

## 5. Conclusions

This study systematically investigated the functions of two immune-related genes, *CTL14* and *PPO4*, in larval development, antiviral immunity, and reproduction of *H. cunea.* Silencing these genes using RNA interference significantly reduced larval survival rates, suppressed larval growth, and weakened host resistance against nucleopolyhedrovirus infection. Moreover, gene silencing caused a severe reduction in adult fecundity and egg hatchability, especially after *CTL14* suppression. Additionally, injection of the purified recombinant immune proteins significantly extended the survival time of virus-challenged larvae. All individuals receiving protein treatment succumbed to viral infection within 16 days, whereas all larvae in the PBS + NPV control group died by day 12. Collectively, our findings demonstrate that *CTL14* and *PPO4* play important roles in both immune defense and developmental physiology in *H. cunea*, highlighting their potential as promising molecular targets for sustainable and environmentally friendly pest control strategies. Future studies should further explore the antiviral functions of *CTL14* and *PPO4* across different insect species and investigate the underlying molecular mechanisms in greater detail.

## Figures and Tables

**Table 1:** Primers used in this study.

Primer Name	Forward Primer Sequences (5′–3′)	Reverse Primer Sequences (5′–3′)	Primer Usage
*HcPPO4*	TCTTCGGAGTAATGGGTGAC	CCGAAGGTATTATTGCCTGC	qRT-PCR
*HcCTL14*	CCTCAGCAGAACAGACGAAA	TGACGGAAATTCTCTGACG	
*EF1-α*	ATGAAATCTCTGTGACCGGGG	GCGGTGTATCGACAAACGT	
*RPL13*	GTTAGCTACACAGCTCCGTGG	GCAGCAGTTGGGGCTTTAGT	
ds*Attacin1*	taatacgactcactataggg ACTTCCAGTTTCAACATCCA	taatacgactcactataggg CTGAGTAGTCCTTACGTTCC	dsRNA synthesis
ds*PPO4*	taatacgactcactataggg CATTCAAGCTATTGAAACCC	taatacgactcactataggg TACGGACAGTTGCCATAACA	
ds*Hdd23-1*	taatacgactcactatagggATAATCGCTGTCTACACAGA	taatacgactcactatagggATGTATTCAAATATGTGTGTGAAG	
ds*CTL14*	taatacgactcactataggg GAAGTAAACTTGGCGAATCC	taatacgactcactataggg CACTGGAATTCACTCTACTG	
ds*GFP*	taatacgactcactataggg GGAGAAGAACTTTTCACTGG	taatacgactcactataggg AGTTGAACGGATCCATCTTC	
*HcPPO4*	atgggtcgcggatccgaattcATGAGCACCCCAAAGGGAGA	gtggtggtggtggtgctcgagCTACCGCTGCCTGGGCAG	Recombinant protein
*HcCTL14*	atgggtcgcgatccgaattcGTTAAGTTTAGATGCGATTATAAATATACTTACC	ctcgagtgcggccgcaagcttCTAACTCGCAGGGACGATATGTT	

Note: Primer usage for dsRNA: lowercase letters represent the T7 promoter. Primer usage for recombinant protein: lowercase letters represent restriction sites.

## Data Availability

The original contributions presented in the study are included in the article, further inquiries can be directed to the corresponding authors.

## Supplementary Materials

The following supporting information can be downloaded at: https://www.mdpi.com/article/10.3390/insects17090885/s1, Figure S1: Analysis of the recombinant CTL14 and PPO4 protein. Left to the right. Lane 1~Lane 4 shows PPO4 protein. Lane 1, 0.6 mM IPTG/16 h at 16 °C; Lane 2, 1 mM IPTG/16 h at 16 °C; Lane 3, 1 mM; Lane 4, 0.6 mM IPTG/16 h at 37 °C; Lane 5, protein marker; Lane 6~Lane9 shows CTL14 protein. Lane 6, 1 mM IPTG/16 h at 37 °C; Lane 7, 0.6 mM IPTG/16 h at 37 °C; Lane 8, 1 mM IPTG/16 h at 16 °C; Lane 9, 0.6 mM IPTG/16 h at 16 °C; Lane 10, CTL14 non-induced. The lane order of the PPO4 samples in this original gel differs from that shown in Figure 9 of the main manuscript. Specifically, Lanes 1 and 2 in Figure 9 correspond to Lanes 4 and 3 of the original gel presented here. The lane arrangement in the main manuscript was adjusted solely to improve figure presentation. No other image processing or data manipulation was performed; Figure S2: Analysis of the recombinant. CTL14 and PPO4 protein. Left to the right. Lane 1, PPO4 non-induced. The image shown in the main manuscript differs from this original gel only in figure arrangement to provide a uniform presentation of the gel panels. No experimental data were altered, and the original uncropped gel is presented here for transparency; Figure S3: Purification of recombinant protein in *E. coli* cells. Left to the right. Lane 4, protein marker; Lane 9, CTL14, 1 mM IPTG/16 h at 16 °C; Lane 10, PPO4, 0.6 mM IPTG/16 h at 16 °C.
