# Supplementary material for: Molecular Cloning and Functional Analysis of CTL14 and PPO4: Their Roles in Development, Reproduction, and Antiviral Defense in Hyphantria cunea
Source: Insects. 2026 Aug 24;17(9):885. doi: 10.3390/insects17090885 (PMC13607632; doi:10.3390/insects17090885)
Supplement: Supplementary file 1 [file insects-17-00885-s001.zip › insects-4394962-supplementary.pdf]

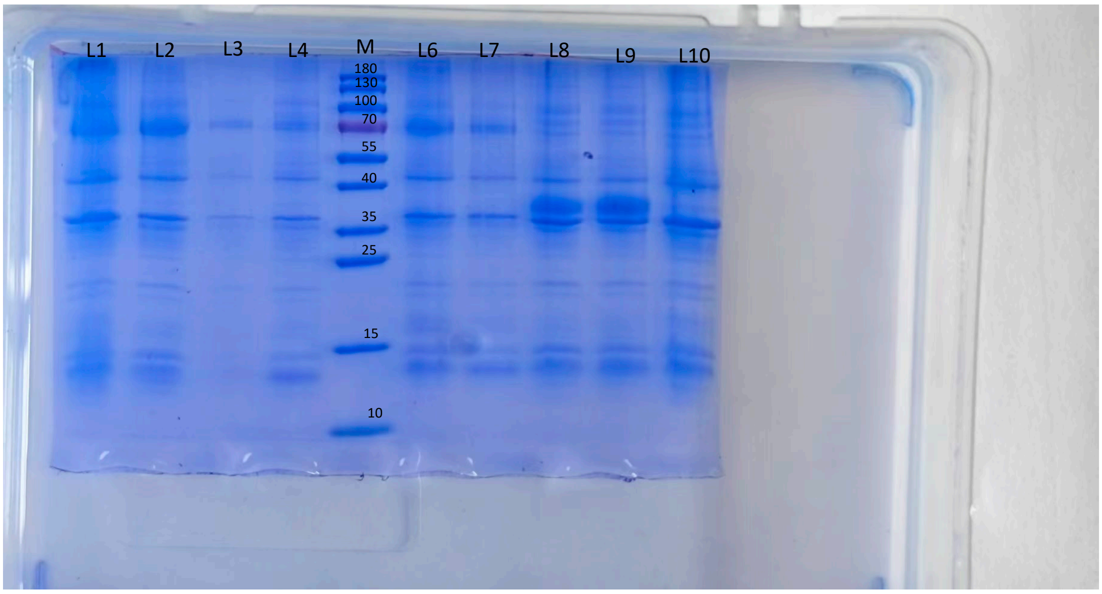

Figure S1: Analysis of the recombinant CTL14 and PPO4 protein. Left to the right. Lane 1~ Lane 4 shows PPO4 protein. Lane 1, 0.6 mM IPTG/16h at 16°C; Lane 2, 1 mM IPTG/16h at 16°C; Lane 3, 1 mM; Lane 4, 0.6 mM IPTG/16h at 37°C; Lane 5, protein marker; Lane 6~Lane9 shows CTL14 protein. Lane 6, 1 mM IPTG/16h at 37°C; Lane 7, 0.6 mM IPTG/16h at 37°C; Lane 8, 1 mM IPTG/16h at 16°C; Lane 9, 0.6 mM IPTG/16h at 16°C; Lane 10, CTL14 non-induced. The lane order of the PPO4 samples in this original gel differs from that shown in Figure 9 of the main manuscript. Specifically, Lanes 1 and 2 in Figure 9 correspond to Lanes 4 and 3 of the original gel presented here. The lane arrangement in the main manuscript was adjusted solely to improve figure presentation. No other image processing or data manipulation was performed.

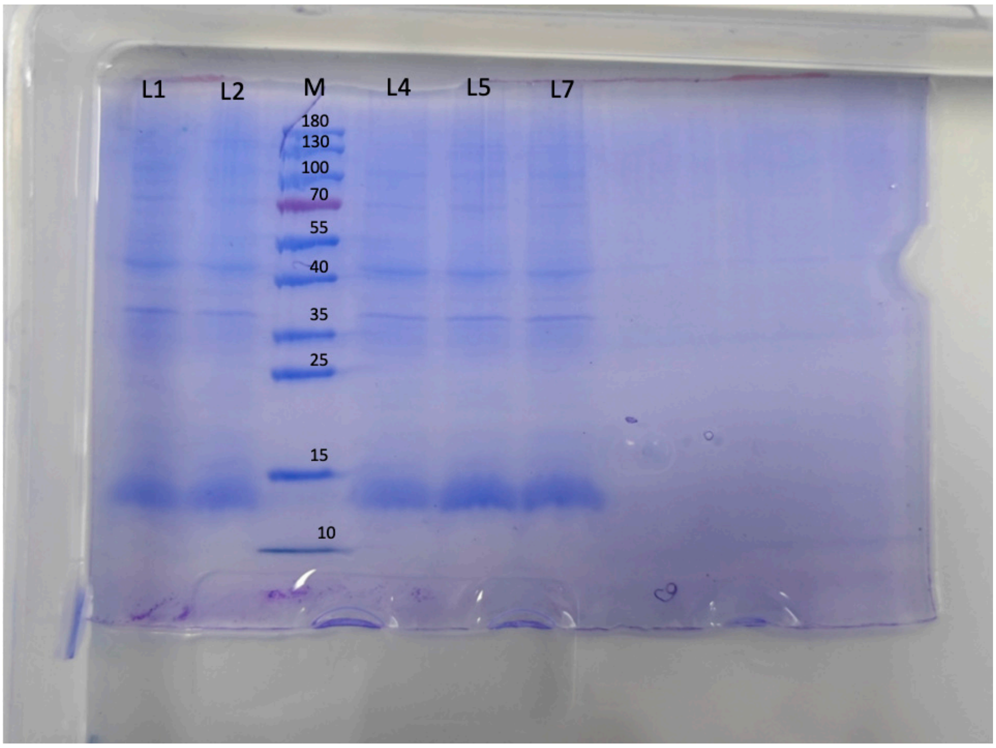

Figure S2: Analysis of the recombinant. CTL14 and PPO4 protein. Left to the right. Lane 1, PPO4 non-induced. The image shown in the main manuscript differs from this original gel only in figure arrangement

to provide a uniform presentation of the gel panels. No experimental data were altered, and the original uncropped gel is presented here for transparency.

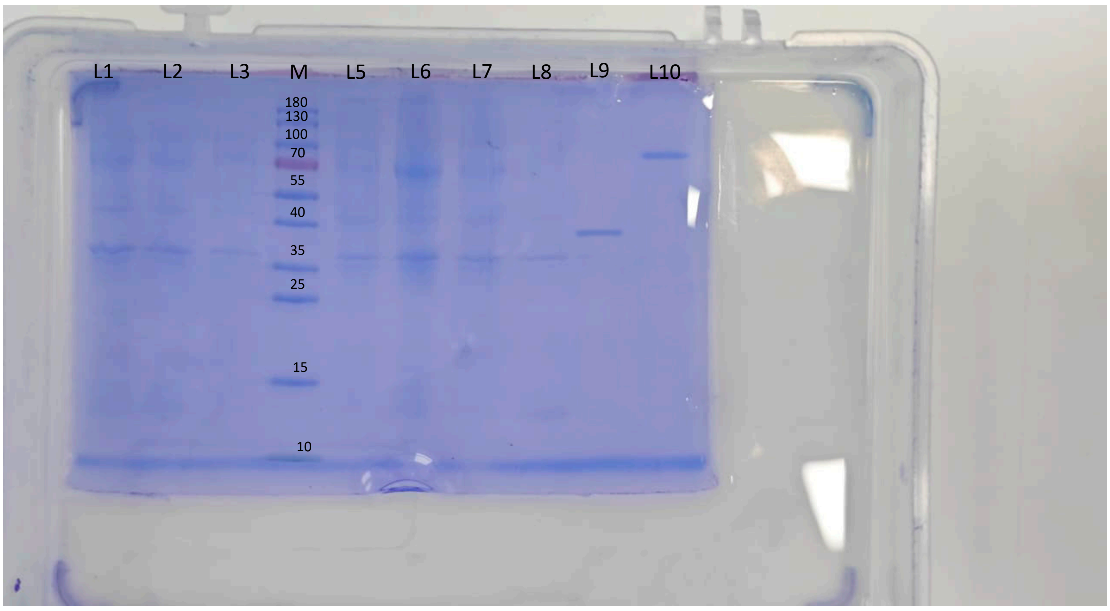

Figure S3: Purification of recombinant protein in *E. coli* cells. Left to the right. Lane 4, protein marker; Lane 9, CTL14, 1 mM IPTG/16h at 16°C; Lane 10, PPO4, 0.6 mM IPTG/16h at 16°C.
